# Human Oncoviruses and p53 Tumor Suppressor Pathway Deregulation at the Origin of Human Cancers

**DOI:** 10.3390/cancers10070213

**Published:** 2018-06-22

**Authors:** Maria Lina Tornesello, Clorinda Annunziata, Anna Lucia Tornesello, Luigi Buonaguro, Franco Maria Buonaguro

**Affiliations:** 1 Molecular Biology and Viral Oncology Unit, Istituto Nazionale Tumori IRCCS “Fondazione G. Pascale”, via Mariano Semmola, 80131 Napoli, Italy; c.annunziata@istitutotumori.na.it (C.A.); a.tornesello@istitutotumori.na.it (A.L.T.); fm.buonaguro@istitutotumori.na.it (F.M.B.); 2 Cancer Immunomodulation Unit, Istituto Nazionale Tumori IRCCS “Fondazione G. Pascale”, via Mariano Semmola, 80131 Napoli, Italy; l.buonaguro@istitutotumori.na.it

**Keywords:** oncoviruses, EBV, HCV, HBV, HPV, HHV-8, HTLVI, MCPyV, p53

## Abstract

Viral oncogenesis is a multistep process largely depending on the complex interplay between viruses and host factors. The oncoviruses are capable of subverting the cell signaling machinery and metabolic pathways and exploit them for infection, replication, and persistence. Several viral oncoproteins are able to functionally inactivate the tumor suppressor p53, causing deregulated expression of many genes orchestrated by p53, such as those involved in apoptosis, DNA stability, and cell proliferation. The Epstein–Barr virus (EBV) BZLF1, the high-risk human papillomavirus (HPV) E6, and the hepatitis C virus (HCV) NS5 proteins have shown to directly bind to and degrade p53. The hepatitis B virus (HBV) HBx and the human T cell lymphotropic virus-1 (HTLV-1) Tax proteins inhibit p53 activity through the modulation of p300/CBP nuclear factors, while the Kaposi’s sarcoma herpesvirus (HHV8) LANA, vIRF-1 and vIRF-3 proteins have been shown to destabilize the oncosuppressor, causing a decrease in its levels in the infected cells. The large T antigen of the Merkel cell polyomavirus (MCPyV) does not bind to p53 but significantly reduces p53-dependent transcription. This review describes the main molecular mechanisms involved in the interaction between viral oncoproteins and p53-related pathways as well as in the development of therapeutic strategies targeting such interactions.

## 1. Introduction

More than 15% of human cancers are caused by infectious agents [1]. Seven human viruses are associated with the majority of pathogen-related tumors, namely, the Epstein–Barr virus (EBV), hepatitis B (HBV) and C (HCV) viruses, human T cell lymphotropic virus 1 (HTLV-1), human papillomaviruses (HPV), Kaposi’s sarcoma herpesvirus (HHV8), and Merkel cell polyomavirus (MCPyV) [2]. Human oncoviruses, although have very different molecular characteristics, share similar features in the mechanisms of tumorigenesis, such as the ability to subvert the host cell signaling pathways involved in the regulation of cell proliferation, genomic stability, differentiation, apoptosis, and recognition by the immune system [3]. In general, human oncoviruses are necessary but not sufficient for full cell transformation. In fact, many people become infected by one or more oncoviruses at some point in their lifetime but only a small fraction of infected subjects eventually develop cancer after decades of persistent infection and viral-related insults to the infected cells. During this long coexistence, multiple genetic and epigenetic alterations accumulate in the chronically infected cells, concurring to the multistage process of cancer development.

The oncogenic activity of tumor viruses mainly relies on their ability to interfere with the p53 signaling cascade in the infected cells [4]. The impairment of oncosuppressors by viral factors was first identified by the study of large and small T antigens encoded by the simian vacuolating virus 40 (SV40) demonstrating their ability to inhibit the activity of tumor suppressors such as p53, pRb, p107, and p130/Rb and to cause evasion of apoptosis [5]. Many in vitro and in vivo studies further corroborated the important aptitude of human viruses to deregulate the activity of p53 [6,7,8]. This review describes several mechanisms used by the seven human oncoviruses to directly or indirectly inactivate p53 function.

## 2. The Tumor Suppressor p53

The p53 protein is a stress sensor regulating many cell pathways, such as cell cycle arrest, senescence, apoptosis, metabolic alterations, DNA repair, and other mechanisms of tumor suppression [9]. Following a cell insult, p53 is rapidly phosphorylated at N-terminus sites by different kinases, including ATM, ATR, CHK1, CHK2, JNK, VRK1, PLK3, and CK1, and the activated protein becomes able to selectively regulate the transcription of more than 200 genes [10,11,12]. Furthermore, the N-terminal-phosphorylated p53 binds to transcriptional coactivators, such as p300 or PCAF, which acetylate the residues at the C-terminus of p53, increasing its stability and binding affinity to the promoters of many genes [13,14]. When the stress response is completed, p53 is reverted to its unphosphorylated form, and its levels are reported to the basal conditions by the activity of the MDM2 protein which ubiquitylates the C-terminal domain of p53 causing its proteasomal degradation [15,16,17]. The interaction between p53 and MDM2 is inhibited by the phosphorylation of p53 at residues Ser15, Thr18, or Ser20 [18].

In the majority of human cancers, p53 activity is often impaired by missense mutations distributed within the exons 4–8 in the *TP53* gene, encoding for the DNA-binding domain [19]. In most cases, mutated p53 proteins become inactive but acquire stability and accumulate in cancer cells [20]. Some mutant p53 proteins, besides losing their oncosuppressor activities, acquire oncogenic features (gain of functions) that endow the cells with overgrowth and survival advantages [21].

The viral-associated tumors rarely harbor mutations in the oncosuppressor proteins. However, in such tumors, the oncogenic viruses interfere with p53 activity by different mechanisms, such as direct binding of viral oncoproteins to p53, phosphorylation of p53 by viral kinases, ubiquitylation, activation of MDM2 expression, which is a negative regulator of p53, or other indirect mechanisms [3].

## 3. The Epstein–Barr Virus (EBV)

The EBV is a herpesvirus with a genome of 184 kb linear double-stranded DNA containing 70 open reading frames (ORFs) coding for latent and lytic proteins. EBV infection is associated with several human malignancies, including endemic Burkitt lymphoma and nasopharyngeal carcinoma [22,23]. The EBV immediate-early transcription factor BZLF1 is the main regulator of the viral life cycle by controlling the switch between its latent and lytic stages [24].

**Figure 1:**
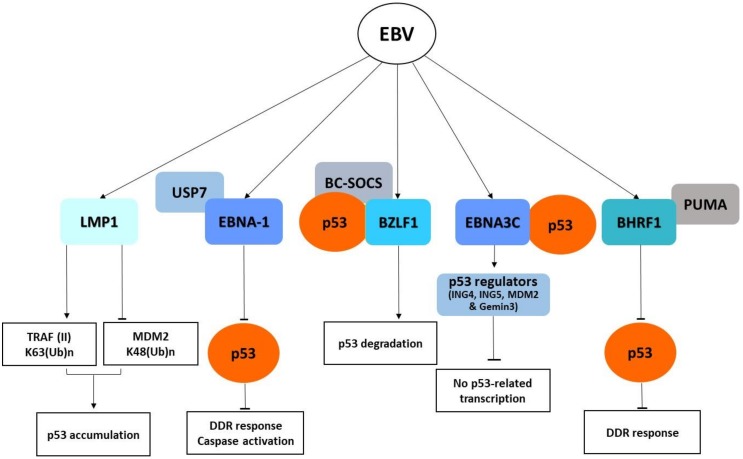
Schematic diagram of Epstein–Barr virus (EBV) oncoproteins affecting p53 signaling pathways. DDR, DNA damage response.

The BZLF1 protein directly interacts with p53 and acts as an adaptor for the elongin BC–cullin 5–SOCS box ubiquitin–protein ligase complex, causing the p53 degradation via a ubiquitin–proteasome pathway independently of MDM2 [25] (Figure 1). Moreover, the viral lytic replication activates the DNA damage response, which causes C-terminus phosphorylation of p53 and further enhances its binding affinity to BZLF1 [25,26].

The EBNA3C protein, produced during the latent phase of EBV life cycle, directly interacts with the C-terminus region of p53, preventing its binding to promoters and the transcription of target genes, and also binds to and stabilizes the p53 regulators ING4, ING5, MDM2, and Gemin3, which inhibit cell apoptosis [27,28].

Following EBV infection, the viral protein EBNA-1 causes genomic instability and at the same time counteracts the DNA damage response (DDR) activation by direct binding to the p53 regulator USP7 and upregulates the survivin protein by inhibiting the downstream caspase activation [29,30]. Interference with the apoptotic pathway is also exerted by two BCL-2 homologues encoded by EBV, namely, BHRF1 and BALF1. The BHRF1 protein has been shown to contribute to the inhibition of p53-dependent DDR signaling by blocking the pro-apoptotic PUMA factor [31].

Moreover, the LMP1 viral protein, which is constitutively expressed in EBV latently infected nasopharyngeal cells, has been shown to promote the accumulation of p53 by two mechanisms: (1) suppression of K48-linked ubiquitination of p53 mediated by the E3 ligase MDM2; (2) induction of K63-linked ubiquitination of p53 through the interaction with tumor necrosis factor receptor-associated factor 2 (TRAF2), which causes p53 accumulation [32].

The current evidence is that EBV deregulates apoptosis by interfering with p53 activity at multiple levels, but further studies are needed to uncover the mechanisms by which EBV causes the full transformation of infected cells.

## 4. The Hepatitis B Virus

The HBV is a small hepadnavirus with a 3.2 kb circular double-stranded DNA genome containing four partial overlapping ORFs encoding the reverse transcriptase/polymerase (Pol), the capsid protein (core antigen HBcAg), three envelope proteins (L, M, and S), and the transactivating protein x (HBx) [32]. HBx is a 154-amino acid protein involved in HBV transcription and viral replication [33].

Many studies have indicated a complex interplay between HBx and p53. HBx is able to physically interact with the C-terminus of p53 and to inhibit its activity by sequestering the oncosuppressors in the cytoplasm [34]. On the other hand, increased levels of p53 have been reported to repress HBx oncogenicity by causing its degradation via overexpression of the negative p53 regulator MDM2 in hepatocellular carcinoma (HCC) [35]. However, the mutated HBx protein exhibits an enhanced inhibitory effect on p53 expression and its downstream signaling compared to wild-type HBx [36]. More recently, it has been reported that HBx causes the overexpression of the long non-coding RNA HUR1 (lnc-HUR1), which in turn interacts with p53 and inhibits the transcriptional regulation of p21 and Bax, thus promoting cell proliferation [37].

Mutations in the *TP53* gene are very frequent in HBV-related HCC. In geographic regions with high prevalence of HBV infection and exposure to aflatoxin B1 (AFB1), the hepatocellular carcinoma DNA often contains a non-synonymous mutation at codon 249 (R249S) in the *TP53* gene [38]. The R249S p53 mutant is able to bind efficiently the HBx and to promote hepatocyte transformation [39].

## 5. The Human T Cell Lymphotropic Virus-1 (HTLV-1)

The HTLV-1 is a retrovirus with a genome of 8.5 kb made of linear, dimeric, single-stranded RNA-positive molecules, containing structural and enzymatic genes as well as overlapping regulatory and accessory genes [40]. The HTLV-1 is recognized as the etiologic agent of adult T-cell leukemia (ALT) and of degenerative, neurological disorders such as tropical spastic paraparesis and HTLV-1-associated myelopathy [41,42]. The two transactivating regulatory proteins Tax and Rex are essential for viral replication. Tax is considered the main oncogenic factor of HTLV-1 for its ability to promote cell cycle entry by CDK activation and to inhibit p53 via the phosphatase Wip1-mediated modulation of p300/CBP nuclear factors [43]. Studies in small animals established that functional inactivation of p53 precedes NF-κB activation and that p53 dysfunction caused by Tax is a critical early event in the onset of Tax-associated leukemia [44].

## 6. The Human Papillomavirus (HPV)

The HPVs are a large and heterogeneous group of small DNA viruses containing 8 Kb circular double-stranded genomes and belong to the Papillomaviridae family. They display a distinct tropism for mucosal (alpha HPVs) or cutaneous (beta and gamma HPVs) squamous epithelia [45].

A group of twelve mucosal HPVs, defined as high-risk viruses, are considered the necessary cause of almost all cervical carcinoma and of a significant fraction of other anogenital and oropharyngeal cancers [46]. The oncogenic activity is mainly due to the ability of early proteins E6 and E7 to subvert the cell cycle control [47,48]. The HPV E6 is a short polypeptide of approximately 150 amino acids consisting of two zinc finger domains which allow the interaction with cellular proteins containing the LXXLL motif [49]. The E6 proteins encoded by high-risk HPVs, but not those of low-risk HPVs, contain a PDZ-binding domain at the C terminus able to bind and degrade cellular targets containing PDZ motifs [50]. The E7 protein encoded by high-risk HPVs is a small protein of approximately 98 amino acids, sharing sequence similarities with the adenovirus E1A protein and SV40 LT antigens [51].

**Figure 2:**
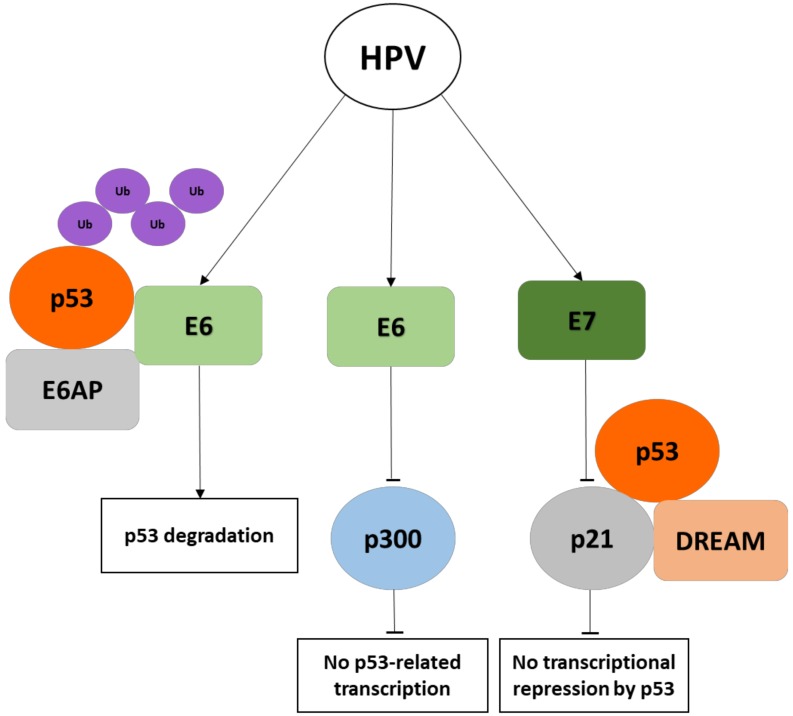
Schematic diagram of human papillomavirus (HPV) E6 and E7 oncoproteins affecting p53 activity. E6AP, E6-associated protein, Ub, ubiquitin, DREAM, DP–RB-like–E2F4–MuvB complex.

The high-risk HPV E6 oncoproteins directly interact with p53 by forming a complex with the E6AP, a cellular E3 ubiquitin ligase, which causes the polyubiquitination and proteasome-mediated degradation of the p53 oncosuppressor [7,52,53]. HPV E6 also promotes the destabilization of p53 by altering the activity of enzymes involved in the modification of p53. Indeed, acetylation of p53, which increases its stability, is required for the responses to DNA damage and for the activation of oncogene checkpoints (Figure 2). The acetylation of p53 at the C-terminus by p300/CBP causes an increase of p53 transcriptional activity leading to growth arrest and apoptosis [14]. High-risk HPV E6 prevents p300-mediated transactivation of p53 through the binding of the histone acetyltransferase at multiple sites [54] and causes the degradation of the histone acetyltransferase hADA3, which is also required for p53-mediated transcriptional activation and apoptosis [55]. The phosphorylation of p53 mediated by ATR prevents its degradation by the MDM2 oncoprotein and activates the p53-dependent DNA repair machinery. However, HPV E6 is able to delay ATR activation with consequent degradation of p53 and abrogation of cell cycle arrest and DNA repair [56]. In addition, HPV E6 has been shown to mask the nuclear localization signal of p53, causing its cytoplasmic retention and decrease of its nuclear transcriptional activity [57].

Cutaneous beta HPVs are involved in skin carcinogenesis [58]. The E6 proteins encoded by beta HPV 38 and HPV 92 have been shown to efficiently interact with p53, inducing its stabilization [59]. Beta HPV 17 E6, despite its weak affinity for p53, is able to cause accumulation of p53 in the cells [59]. An additional study showed that HPV 49 E6, similarly to the high-risk mucosal HPV E6, binds and promotes the degradation of p53 via the E6AP-dependent proteasome pathway [60]. Moreover, the E6 proteins encoded by HPV 5 and HPV 8 interact with and cause p300 degradation via the proteasome pathway, which is activated by a decreased S1834 phosphorylation at the C-terminus of p53 [61]. The interaction of cottontail rabbit papillomavirus (CRPV) E6 and HPV 38 E6 with the histone acetyltransferase p300 causes the block of p53-mediated apoptosis [62]. In fact, the E6 mutants which are unable to bind p300 do not inhibit p53 acetylation, p53-dependent transcription, and apoptosis [62].

The E7 proteins encoded by high-risk HPVs are capable of deregulating p53-mediated cell cycle arrest by interfering with the cyclin-dependent kinase inhibitor p21 (CDKN1A), which represents a key mediator of p53 checkpoint control [63,64,65]. The HPV E7 oncoprotein has also been shown to disrupt the pRB-related transcriptional repressor complex DREAM (DP, RB-like, E2F4, and MuvB), which is an important effector of cell cycle checkpoint activation mediated by the p53–p21–DREAM pathway [66,67]). The disruption of such complex by E7 abrogates p53-dependent downregulation of DREAM, causing increased expression of a large number of cell cycle genes and impairment of cell cycle checkpoints [68,69].

## 7. The Hepatitis C Virus

The HCV of the flaviviridae family has a single-stranded RNA genome encoding a 3000-amino acid polyprotein which is cleaved by viral and cellular proteases into four structural proteins, named capsid protein C, envelope glycoproteins E1 and E2, and protein P7, and in six nonstructural proteins, named NS2, NS3, NS4A, NS4B, NS5A, and NS5B, [70]. HCV is the cause of hepatocellular carcinoma and lymphoproliferative disorders.

The core protein of HCV directly binds the C-terminus of p53 causing the increase of p53 transcriptional activity and elevated expression of p21 (CDKN1A), a major target of p53 [71]. In addition, the core protein interacts also with hTAF(II), a component of the transcriptional factor complex and coactivator of p53, suggesting that it widely modulates promoter activities during HCV infection [71].

HCV NS2 has been shown to interfere with the DNA damage checkpoint pathway by causing retention of p53 in the cytoplasm and favoring cell proliferation [72].

HCV NS5A has been shown to bind directly to p53 and hTAF(II), to partially sequester them in the cytoplasm, and to suppress p53-mediated transcriptional transactivation and apoptosis during HCV infection [73]. Further studies by microarray analysis showed that HCV NS5A causes the downregulation of nine genes, including *TP53*, and the upregulation of 10 genes, including those coding for survivin, NOS2A, cyclin D1, and NF-κB, which are all associated with signal transduction [73]. The inhibition of p53 by HCV NS5A has also been shown to interfere with the DNA damage checkpoint pathway by causing the downregulation of the growth arrest and DNA-damage-inducible gene 45-α (GADD45α) [74]. The use of pharmacological inhibitors or small interfering RNAs can reverse NS5A-mediated downregulation of p53 and GADD45α, suggesting that NS5A is a main contributor to liver carcinogenesis [74].

## 8. The Kaposi’s Sarcoma Herpesvirus (HHV8)

The HHV8 is a human gamma herpesvirus with a linear double-strand DNA genome of approximately 165 kb containing 100 ORFs. The virus has a sequence homology to the closely related EBV and unique genes not similar to any other herpesvirus [75]. The HHV8 is recognized as the causative agent of three different types of malignancies, i.e., Kaposi’s sarcoma (KS), multicentric Castelman’s disease, and a form of AIDS-related primary effusion lymphoma (PEL) [76,77].

Nearly 25 unique genes may represent captured and diverged homologues of cellular genes that are referred to as ORF-K. A number of proteins encoded by conserved or unique genes have been suggested to be responsible for KS pathogenesis: K1, K2, vMIPS, K4, K4.1, K5, K9, K12, ORF-6, ORF-71, ORF-72, ORF-73, ORF-74, and K15 [78]. Among these, LANA and v-cyclin, encoded by ORF-72 and ORF-73, respectively, have demonstrated to affect cell cycle checkpoint mediators. The LANA protein acts as an adaptor molecule for an E3 ubiquitin complex via a specific protein motif and causes ubiquitylation and degradation of p53 [79]. LANA inhibits both p53 and pRB tumor suppressor pathways, allowing the infected cells to become resistant to anti-growth signals and cell cycle arrest and to accumulate genetic damages [80,81].

ORF K9 codes for a viral homolog of interferon inducible factor vIRF-1, which inhibits the tumor suppressor p53 via the interaction with its co-activator p300/CBP and promotes its ubiquitination and delocalization to the cytoplasm [82]. In addition, vIRF-1 can prevent apoptosis during viral replication by direct inhibition of pro-apoptotic BH3 domain-containing proteins such as BIM [83]. The protein vIRF-1 is also able to inhibit the activation of p53 by ATM, causing suppression of apoptosis induced by DNA damage [84]. The virus-encoded viral interferon regulatory factor 3 (vIRF-3) gene is a latent gene which is involved in the regulation of apoptosis, cell cycle, antiviral immunity, and tumorigenesis. In addition, vIRF-3 has been shown to interact with the DNA-binding domain of p53, to inhibit p53 phosphorylation on serine residues S15 and S20, and to destabilize the oncosuppressor by increasing its polyubiquitination and proteasome-mediated degradation [85].

**Figure 3:**
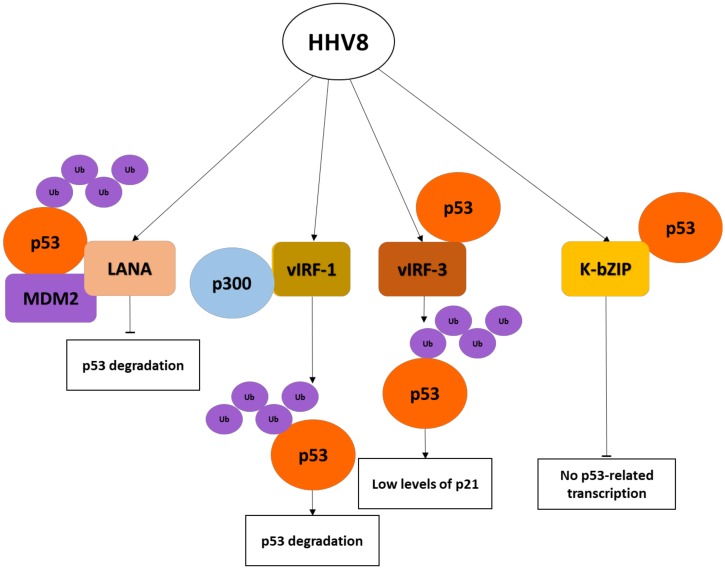
Schematic diagram of Kaposi’s sarcoma herpesvirus (HHV8) oncoproteins affecting p53 signaling pathways. K-bZIP, basic region–leucine zipper.

The K-bZIP protein encoded by ORF K8 has a basic region–leucine zipper and interacts with the DNA-binding region of p53, causing inhibition of p53-dependent transcription [86]. Other viral proteins, such as the lytic protein vIRF4, lack E3 ligase activity but they are capable of forming multi-protein complexes with ubiquitin ligase activity and facilitate the proteasome-mediated degradation of p53 [65]. Thus, HHV8 has several efficient mechanisms to downregulate p53 function and facilitate uncontrolled cell proliferation and tumor growth (Figure 3).

## 9. The Merkel Cell Polyomavirus (MCPyV)

The MCPyV is a polyomavirus with a double-stranded circular DNA genome of 5.4 kb. The virus codes for three early viral transcripts, namely, the large T antigen (LT), small T antigen (sT), and 57KT, which are produced by alternative splicing of T early region, and for two structural capsid proteins (VP1 and VP2) which are coded by the late region of the viral genome [87]. The virus is associated with the development of Merkel cell carcinoma (MCC) which is the most aggressive skin cancer in humans [88].

The MCPyV LT has shown to play a key role in the viral life cycle as well as in carcinogenesis [89,90]. The full-length MCPyV LT protein does not bind directly p53 but causes a reduction of p53-dependent transcription in reporter assays [91]. The truncated LT protein, expressed in the majority of MCPyV-related tumours, does not bind to p53 or reduce p53-dependent transcription but shows very high binding affinity for Rb and is able to partially relocalize Rb to the cytoplasm [91].

## 10. Targeting the p53 Proteasome in Virus-Related Human Cancers

The levels of p53 are tightly controlled by the cellular antagonist MDM2 which regulates the stability and activity of p53 by an E3-ubiquitin ligase activity [92]. The crystal structure of p53 in complex with MDM2 and MDMX revealed that only three p53 amino acid residues (Phe19, Trp23, and Leu26) contribute extensively to the physical interaction between p53 and MDM2/MDMX proteins [93].

A variety of compounds able to bind the N-terminal pocket of MDM2 and to disrupt the MDM2–p53 interaction have been developed and offer new therapeutic opportunities for tumors harboring wild-type p53 [94]. The most promising results have been obtained with the use of Nutlin-3, a small cis-imidazoline analogue competing with MDM2 for binding to p53 [95]. Several in vitro and in vivo studies have demonstrated that Nutlin-3 is able to selectively enhance apoptosis in cancer cells containing wild-type p53 [96]. Nutlin-3 is non-genotoxic and protects normal cells from mitotic toxicity [96]. Moreover, Nutlin-3 can activate the p53 pathway and sensitize EBV-positive nasopharyngeal carcinoma cells to cisplatin-induced apoptosis [97] The use of Nutlin-3 in Burkitt lymphoma cells chronically infected with EBV has been shown to disrupt the interaction of p53 with MDM2 and to facilitate apoptosis [98]. Similar results have been observed by treating EBV-positive lymphoblastoid cells with Nutlin-3, which caused p53 reactivation and apoptosis [99].

In addition, the restoration of the p53 pathway with Nutlin-3 has been shown to cause a specific and highly potent activation of cell death in HHV8-related pleural effusion lymphomas [100]. Such results demonstrated that Nutlin-3 is able to disrupt the p53–MDM2–LANA complex and to suppress the anti-apoptotic activity caused by the complex created by LANA, p53, and MDM2 [100,101,102].

Similarly to Nutilin-3, several other small molecule inhibitors able to bind MDM2 and to inhibit its association with p53 have been developed and evaluated in preclinical and clinical studies [103]. Such therapeutic compounds include cis-imidazoline (NCT00559533, NCT01635296, NCT01605526, NCT01462175, NCT01773408), spiro-oxindole (NCT01636479, NCT01985191), imidazothiazole (NCT01877382), dihydroisoquinolinone (NCT01760525), piperidines (NCT01451437), piperidinone (NCT01723020, NCT02110355) and pyrrolidine (NCT02407080) molecules which are in phase I clinical trials for the treatment of several solid tumors [103].

Different inhibitors targeting the p53–MDM2 interaction, based on phage display peptide libraries [104] or on the designed sequences of stapled peptides derived from the α-helix of the p53 transactivation domain [105], have been developed. Indeed, the restoration of p53 activity has been achieved by targeting MDMX with the stapled peptide SAH-p53-8, which blocks the formation of the p53–MDMX complex and restores the transcriptional upregulation of p53-related genes and the reduction of tumor cell viability [106]. The dual inhibition of MDM2/MDMX with the stapled α-helical peptide ALRN-6924 (Aileron Therapeutics, Cambridge, MA, USA) has been demonstrated to strongly activate p53-dependent transcription and to produce a marked antileukemic effect [107]. Moreover, ALRN-6924 showed promising pre-clinical activity in cell lines harboring wild-type p53 and in patient-derived xenograft models, as well as induced complete remission in a patient with an angioimmunoblastic T-cell lymphoma containing wild-type p53 [108]. This compound has recently entered phase I clinical testing (NCT02264613).

In HPV-related cancers, E6-mediated degradation of p53 represents an important mechanism of cell transformation [109]. The E6 proteins of high-risk HPVs interact with the LxxLL motif of the cellular ubiquitin ligase E6AP, which leads to the recruitment and polyubiquitination of p53 [110]. The x-ray structure of the HPV16 E6–E6AP complex showed a distinct binding pocket for E6AP in the E6 protein. This pocket provides a docking site for peptide inhibitors such as pep11** which binds to HPV16 E6 and induces apoptosis of HPV16-positive cancer cells [111]. The binding of pep11** blocks E6-mediated p53 degradation by capturing E6 in trimeric E6–pep11**–p53 complexes [112].

In addition, the binding of the E6AP LxxLL-derived peptide to the E6 oncoprotein renders the conformation of E6 competent for interaction with p53 and causes the formation of a p53-binding cleft on the E6 [7]. The p53-binding site on E6 may represent a second potential binding site for peptide inhibitors to be used in combination strategies targeting both the LxxLL pocket and the p53-binding cleft on E6 to obtain an efficient disruption of the E6–E6AP–p53 complex [7].

The further development of specific molecules targeting the interaction of p53 with viral oncoproteins might provide promising strategies to interfere with virus-related tumorigenesis.

## 11. Conclusions

The development of virus-associated cancer depends on the complex interplay between host and viral factors. The seven recognized human oncoviruses encode regulatory proteins that subvert cell signaling and modulate growth and differentiation. The functional impairment of p53 activity is a crucial mechanism of viral-related carcinogenesis. Several viral proteins have been shown to interact directly or indirectly with p53, facilitating MDM2/MDMX-mediated degradation. The study of the crystallographic structure of p53 and of its interactions with HPV E6 and cellular proteins has provided the basis for the development of specific inhibitors of p53 degradation. Several pharmaceutical companies have developed drugs targeting the negative regulators of p53, such as MDM2 and MDMX, that are in preclinical study and into early-phase clinical trials. Further studies are needed to establish whether similar structural propensities exist within other viral proteins that interact with p53 and its regulators.

## References

[1] Plummer M., de Martel C., Vignat J., Ferlay J., Bray F., Franceschi S. (2016). Global burden of cancers attributable to infections in 2012: A synthetic analysis. Lancet Glob. Health.

[2] Bouvard V., Baan R., Straif K., Grosse Y., Secretan B., El Ghissassi F., Benbrahim-Tallaa L., Guha N., Freeman C., Galichet L. (2009). A review of human carcinogens—Part B: Biological agents. Lancet Oncol..

[3] Mesri E.A., Feitelson M.A., Munger K. (2014). Human viral oncogenesis: A cancer hallmarks analysis. Cell Host. Microbe.

[4] Tornesello M.L., Buonaguro L., Buonaguro F.M. (2015). An overview of new biomolecular pathways in pathogen-related cancers. Futur. Oncol..

[5] Saenz-Robles M.T., Sullivan C.S., Pipas J.M. (2001). Transforming functions of Simian Virus 40. Oncogene.

[6] Lazo P.A., Santos C.R. (2011). Interference with p53 functions in human viral infections, a target for novel antiviral strategies?. Rev. Med. Virol..

[7] Martinez-Zapien D., Ruiz F.X., Poirson J., Mitschler A., Ramirez J., Forster A., Cousido-Siah A., Masson M., Pol S.V., Podjarny A. (2016). Structure of the E6/E6AP/p53 complex required for HPV-mediated degradation of p53. Nature.

[8] Aloni-Grinstein R., Charni-Natan M., Solomon H., Rotter V. (2018). p53 and the Viral Connection: Back into the Future (double dagger). Cancers.

[9] Bieging K.T., Mello S.S., Attardi L.D. (2014). Unravelling mechanisms of p53-mediated tumour suppression. Nat. Rev. Cancer.

[10] Meek D.W., Anderson C.W. (2009). Posttranslational modification of p53: Cooperative integrators of function. Cold Spring Harb. Perspect. Biol..

[11] Teufel D.P., Bycroft M., Fersht A.R. (2009). Regulation by phosphorylation of the relative affinities of the N-terminal transactivation domains of p53 for p300 domains and Mdm2. Oncogene.

[12] Lee C.W., Ferreon J.C., Ferreon A.C., Arai M., Wright P.E. (2010). Graded enhancement of p53 binding to CREB-binding protein (CBP) by multisite phosphorylation. Proc. Natl. Acad. Sci. USA.

[13] Sakaguchi K., Herrera J.E., Saito S., Miki T., Bustin M., Vassilev A., Anderson C.W., Appella E. (1998). DNA damage activates p53 through a phosphorylation-acetylation cascade. Genes Dev..

[14] Reed S.M., Quelle D.E. (2014). p53 Acetylation: Regulation and Consequences. Cancers.

[15] Brooks C.L., Gu W. (2006). p53 ubiquitination: Mdm2 and beyond. Mol. Cell.

[16] Iwakuma T., Lozano G. (2003). MDM2, an introduction. Mol. Cancer Res..

[17] Perry M.E. (2010). The regulation of the p53-mediated stress response by MDM2 and MDM4. Cold Spring Harb. Perspect. Biol..

[18] Kussie P.H., Gorina S., Marechal V., Elenbaas B., Moreau J., Levine A.J., Pavletich N.P. (1996). Structure of the MDM2 oncoprotein bound to the p53 tumor suppressor transactivation domain. Science.

[19] Soussi T. (2011). TP53 mutations in human cancer: Database reassessment and prospects for the next decade. Adv. Cancer Res..

[20] Rivlin N., Brosh R., Oren M., Rotter V. (2011). Mutations in the p53 Tumor Suppressor Gene: Important Milestones at the Various Steps of Tumorigenesis. Genes Cancer.

[21] Zhang Y., Coillie S.V., Fang J.Y., Xu J. (2016). Gain of function of mutant p53: R282W on the peak?. Oncogenesis.

[22] Klein G. (2015). Tumor Associations of EBV—Historical Perspectives. Curr. Top. Microbiol. Immunol..

[23] Fitzsimmons L., Kelly G.L. (2017). EBV and Apoptosis: The Viral Master Regulator of Cell Fate?. Viruses.

[24] Chevallier-Greco A., Manet E., Chavrier P., Mosnier C., Daillie J., Sergeant A. (1986). Both Epstein-Barr virus (EBV)-encoded trans-acting factors, EB1 and EB2, are required to activate transcription from an EBV early promoter. EMBO J..

[25] Sato Y., Kamura T., Shirata N., Murata T., Kudoh A., Iwahori S., Nakayama S., Isomura H., Nishiyama Y., Tsurumi T. (2009). Degradation of phosphorylated p53 by viral protein-ECS E3 ligase complex. PLoS Pathog..

[26] Sato Y., Shirata N., Kudoh A., Iwahori S., Nakayama S., Murata T., Isomura H., Nishiyama Y., Tsurumi T. (2009). Expression of Epstein-Barr virus BZLF1 immediate-early protein induces p53 degradation independent of MDM2, leading to repression of p53-mediated transcription. Virology.

[27] Cai Q., Guo Y., Xiao B., Banerjee S., Saha A., Lu J., Glisovic T., Robertson E.S. (2011). Epstein-Barr virus nuclear antigen 3C stabilizes Gemin3 to block p53-mediated apoptosis. PLoS. Pathog..

[28] Yi F., Saha A., Murakami M., Kumar P., Knight J.S., Cai Q., Choudhuri T., Robertson E.S. (2009). Epstein-Barr virus nuclear antigen 3C targets p53 and modulates its transcriptional and apoptotic activities. Virology.

[29] Saridakis V., Sheng Y., Sarkari F., Holowaty M.N., Shire K., Nguyen T., Zhang R.G., Liao J., Lee W., Edwards A.M. (2005). Structure of the p53 binding domain of HAUSP/USP7 bound to Epstein-Barr nuclear antigen 1 implications for EBV-mediated immortalization. Mol. Cell.

[30] Lu J., Murakami M., Verma S.C., Cai Q., Haldar S., Kaul R., Wasik M.A., Middeldorp J., Robertson E.S. (2011). Epstein-Barr Virus nuclear antigen 1 (EBNA1) confers resistance to apoptosis in EBV-positive B-lymphoma cells through up-regulation of survivin. Virology.

[31] Kvansakul M., Wei A.H., Fletcher J.I., Willis S.N., Chen L., Roberts A.W., Huang D.C.S., Colman P.M. (2010). Structural basis for apoptosis inhibition by Epstein-Barr virus BHRF1. PLoS. Pathog..

[32] Li L., Li W., Xiao L., Xu J., Chen X., Tang M., Dong Z., Tao Q., Cao Y. (2012). Viral oncoprotein LMP1 disrupts p53-induced cell cycle arrest and apoptosis through modulating K63-linked ubiquitination of p53. Cell Cycle.

[33] McClain S.L., Clippinger A.J., Lizzano R., Bouchard M.J. (2007). Hepatitis B virus replication is associated with an HBx-dependent mitochondrion-regulated increase in cytosolic calcium levels. J. Virol..

[34] Soria C., Estermann F.E., Espantman K.C., O'Shea C.C. (2010). Heterochromatin silencing of p53 target genes by a small viral protein. Nature.

[35] Xian L., Zhao J., Wang J., Fang Z., Peng B., Wang W., Ji X., Yu L. (2010). p53 Promotes proteasome-dependent degradation of oncogenic protein HBx by transcription of MDM2. Mol. Biol. Rep..

[36] Iyer S., Groopman J.D. (2011). Interaction of mutant hepatitis B X protein with p53 tumor suppressor protein affects both transcription and cell survival. Mol. Carcinog..

[37] Liu N., Liu Q., Yang X., Zhang F., Li X., Ma Y., Guan F., Zhao X., Li Z., Zhang L. (2018). Hepatitis B virus-upregulated lnc-HUR1 promotes cell proliferation and tumorigenesis by blocking p53 activity. Hepatology.

[38] Tornesello M.L., Buonaguro L., Tatangelo F., Botti G., Izzo F., Buonaguro F.M. (2013). Mutations in TP53, CTNNB1 and PIK3CA genes in hepatocellular carcinoma associated with hepatitis B and hepatitis C virus infections. Genomics.

[39] Gouas D.A., Shi H., Hautefeuille A.H., Ortiz-Cuaran S.L., Legros P.C., Szymanska K.J., Galy O., Egevad L.A., Abedi-Ardekani B., Wiman K.G. (2010). Effects of the TP53 p.R249S mutant on proliferation and clonogenic properties in human hepatocellular carcinoma cell lines: Interaction with hepatitis B virus X protein. Carcinogenesis.

[40] Younis I., Yamamoto B., Phipps A., Green P.L. (2005). Human T-cell leukemia virus type 1 expressing nonoverlapping tax and rex genes replicates and immortalizes primary human T lymphocytes but fails to replicate and persist in vivo. J. Virol..

[41] Kannian P., Green P.L. (2010). Human T Lymphotropic Virus Type 1 (HTLV-1): Molecular Biology and Oncogenesis. Viruses.

[42] Derse D., Hill S.A., Lloyd P.A., Chung H., Morse B.A. (2001). Examining human T-lymphotropic virus type 1 infection and replication by cell-free infection with recombinant virus vectors. J. Virol..

[43] Zane L., Yasunaga J., Mitagami Y., Yedavalli V., Tang S.-W., Chen C.-Y., Ratner L., Lu X., Jeang K.-T. (2012). Wip1 and p53 contribute to HTLV-1 Tax-induced tumorigenesis. Retrovirology.

[44] Ohsugi T., Ishida T., Shimasaki T., Okada S., Umezawa K. (2013). p53 dysfunction precedes the activation of nuclear factor-kappaB during disease progression in mice expressing Tax, a human T-cell leukemia virus type 1 oncoprotein. Carcinogenesis.

[45] De Villiers E.M., Fauquet C., Broker T.R., Bernard H.U., zur Hausen H. (2004). Classification of papillomaviruses. Virology.

[46] De Martel C., Plummer M., Vignat J., Franceschi S. (2017). Worldwide burden of cancer attributable to HPV by site, country and HPV type. Int. J. Cancer.

[47] Moody C. (2017). Mechanisms by which HPV Induces a Replication Competent Environment in Differentiating Keratinocytes. Viruses.

[48] Tommasino M. (2014). The human papillomavirus family and its role in carcinogenesis. Semin. Cancer Biol..

[49] Zanier K., Charbonnier S., Sidi A.O., McEwen A.G., Ferrario M.G., Poussin-Courmontagne P., Cura V., Brimer N., Babah K.O., Ansari T. (2013). Structural basis for hijacking of cellular LxxLL motifs by papillomavirus E6 oncoproteins. Science.

[50] Ganti K., Broniarczyk J., Manoubi W., Massimi P., Mittal S., Pim D., Szalmas A., Thatte J., Thomas M., Tomaić V. (2015). The Human Papillomavirus E6 PDZ Binding Motif: From Life Cycle to Malignancy. Viruses.

[51] Phelps W.C., Yee C.L., Munger K., Howley P.M. (1988). The human papillomavirus type 16 E7 gene encodes transactivation and transformation functions similar to those of adenovirus E1A. Cell.

[52] Hoppe-Seyler K., Bossler F., Braun J.A., Herrmann A.L., Hoppe-Seyler F. (2018). The HPV E6/E7 Oncogenes: Key Factors for Viral Carcinogenesis and Therapeutic Targets. Trends Microbiol..

[53] Katzenellenbogen R.A., Vliet-Gregg P., Xu M., Galloway D.A. (2009). NFX1-123 increases hTERT expression and telomerase activity posttranscriptionally in human papillomavirus type 16 E6 keratinocytes. J. Virol..

[54] Xie X., Piao L., Bullock B.N., Smith A., Su T., Zhang M., Teknos T.N., Arora P.S., Pan Q. (2014). Targeting HPV16 E6-p300 interaction reactivates p53 and inhibits the tumorigenicity of HPV-positive head and neck squamous cell carcinoma. Oncogene.

[55] Chand V., John R., Jaiswal N., Johar S.S., Nag A. (2014). High-risk HPV16E6 stimulates hADA3 degradation by enhancing its SUMOylation. Carcinogenesis.

[56] Wallace N.A., Galloway D.A. (2015). Novel Functions of the Human Papillomavirus E6 Oncoproteins. Annu. Rev. Virol..

[57] Mantovani F., Banks L. (2001). The human papillomavirus E6 protein and its contribution to malignant progression. Oncogene.

[58] Tommasino M. (2017). The biology of beta human papillomaviruses. Virus Res..

[59] White E.A., Kramer R.E., Tan M.J., Hayes S.D., Harper J.W., Howley P.M. (2012). Comprehensive analysis of host cellular interactions with human papillomavirus E6 proteins identifies new E6 binding partners and reflects viral diversity. J. Virol..

[60] Cornet I., Bouvard V., Campo M.S., Thomas M., Banks L., Gissmann L., Lamartine J., Sylla B.S., Accardi R., Tommasino M. (2012). Comparative analysis of transforming properties of E6 and E7 from different beta human papillomavirus types. J. Virol..

[61] Howie H.L., Koop J.I., Weese J., Robinson K., Wipf G., Kim L., Galloway D.A. (2011). Beta-HPV 5 and 8 E6 promote p300 degradation by blocking AKT/p300 association. PLoS. Pathog..

[62] Muench P., Probst S., Schuetz J., Leiprecht N., Busch M., Wesselborg S., Stubenrauch F., Iftner T. (2010). Cutaneous papillomavirus E6 proteins must interact with p300 and block p53-mediated apoptosis for cellular immortalization and tumorigenesis. Cancer Res..

[63] Songock W.K., Kim S.M., Bodily J.M. (2017). The human papillomavirus E7 oncoprotein as a regulator of transcription. Virus Res..

[64] Demers G.W., Foster S.A., Halbert C.L., Galloway D.A. (1994). Growth arrest by induction of p53 in DNA damaged keratinocytes is bypassed by human papillomavirus 16 E7. Proc. Natl. Acad. Sci. USA.

[65] Lee H.R., Toth Z., Shin Y.C., Lee J.-S., Chang H., Gu W., Oh T.-K., Kim M.H., Jung J.U. (2009). Kaposi’s sarcoma-associated herpesvirus viral interferon regulatory factor 4 targets MDM2 to deregulate the p53 tumor suppressor pathway. J. Virol..

[66] Nor Rashid N., Yusof R., Watson R.J. (2011). Disruption of repressive p130-DREAM complexes by human papillomavirus 16 E6/E7 oncoproteins is required for cell-cycle progression in cervical cancer cells. J. Gen. Virol..

[67] Sadasivam S., DeCaprio J.A. (2013). The DREAM complex: Master coordinator of cell cycle-dependent gene expression. Nat. Rev. Cancer.

[68] Fischer M., Uxa S., Stanko C., Magin T.M., Engeland K. (2017). Human papilloma virus E7 oncoprotein abrogates the p53-p21-DREAM pathway. Sci. Rep..

[69] Engeland K. (2018). Cell cycle arrest through indirect transcriptional repression by p53: I. have a DREAM. Cell Death. Differ..

[70] Chevaliez S., Pawlotsky J.-M., Tan S.L. (2006). HCV Genome and Life Cycle. Hepatitis C Viruses: Genomes and Molecular Biology.

[71] Otsuka M., Kato N., Lan K., Yoshida H., Kato J., Goto T., Shiratori Y., Omata M. (2000). Hepatitis C virus core protein enhances p53 function through augmentation of DNA binding affinity and transcriptional ability. J. Biol. Chem..

[72] Bittar C., Shrivastava S., Bhanja C.J., Rahal P., Ray R.B. (2013). Hepatitis C virus NS2 protein inhibits DNA damage pathway by sequestering p53 to the cytoplasm. PLoS ONE.

[73] Lan K.H., Sheu M.L., Hwang S.J., Yen S.-H., Chen S.-Y., Wu J.-C., Wang Y.-J., Kato N., Omata M., Chang F.-Y. (2002). HCV NS5A interacts with p53 and inhibits p53-mediated apoptosis. Oncogene.

[74] Cheng D., Zhao L., Zhang L., Jiang Y., Tian Y., Xiao X., Gong G. (2013). p53 controls hepatitis C virus non-structural protein 5A-mediated downregulation of GADD45α expression via the NF-κB and PI3K-Akt pathways. J. Gen. Virol..

[75] Russo J.J., Bohenzky R.A., Chien M.C., Chen J., Yan M., Maddalena D., Parry J.P., Peruzzi D., Edelman I.S., Chang Y. (1996). Nucleotide sequence of the Kaposi sarcoma-associated herpesvirus (HHV8). Proc. Natl. Acad. Sci. USA.

[76] Chang Y., Cesarman E., Pessin M.S., Lee F., Culpepper J., Knowles D.M., Moore P.S. (1994). Identification of herpesvirus-like DNA sequences in AIDS-associated Kaposi’s sarcoma. Science.

[77] Cesarman E., Chang Y., Moore P.S., Said J.W., Knowles D.M. (1995). Kaposi's sarcoma-associated herpesvirus-like DNA sequences in AIDS-related body-cavity-based lymphomas. N. Engl. J. Med..

[78] Neipel F., Fleckenstein B. (1999). The role of HHV-8 in Kaposi's sarcoma. Semin. Cancer Biol..

[79] Suzuki T., Isobe T., Kitagawa M., Ueda K. (2010). Kaposi's sarcoma-associated herpesvirus-encoded LANA positively affects on ubiquitylation of p53. Biochem. Biophys. Res. Commun..

[80] Radkov S.A., Kellam P., Boshoff C. (2000). The latent nuclear antigen of Kaposi sarcoma-associated herpesvirus targets the retinoblastoma-E2F pathway and with the oncogene Hras transforms primary rat cells. Nat. Med..

[81] Si H., Robertson E.S. (2006). Kaposi's sarcoma-associated herpesvirus-encoded latency-associated nuclear antigen induces chromosomal instability through inhibition of p53 function. J. Virol..

[82] Nakamura H., Li M., Zarycki J., Jung J.U. (2001). Inhibition of p53 tumor suppressor by viral interferon regulatory factor. J. Virol..

[83] Choi Y.B., Nicholas J. (2010). Bim nuclear translocation and inactivation by viral interferon regulatory factor. PLoS Pathog..

[84] Shin Y.C., Nakamura H., Liang X., Feng P., Chang H., Kowalik T.F., Jung J.U. (2006). Inhibition of the ATM/p53 signal transduction pathway by Kaposi’s sarcoma-associated herpesvirus interferon regulatory factor 1. J. Virol..

[85] Baresova P., Musilova J., Pitha P.M., Lubyova B. (2014). p53 tumor suppressor protein stability and transcriptional activity are targeted by Kaposi's sarcoma-associated herpesvirus-encoded viral interferon regulatory factor 3. Mol. Cell Biol..

[86] Park J., Seo T., Hwang S., Lee D., Gwack Y., Choe J. (2000). The K-bZIP protein from Kaposi's sarcoma-associated herpesvirus interacts with p53 and represses its transcriptional activity. J. Virol..

[87] Shuda M., Feng H., Kwun H.J., Rosen S.T., Gjoerup O., Moore P.S., Chang Y. (2008). T antigen mutations are a human tumor-specific signature for Merkel cell polyomavirus. Proc. Natl. Acad. Sci. USA.

[88] Feng H., Shuda M., Chang Y., Moore P.S. (2008). Clonal integration of a polyomavirus in human Merkel cell carcinoma. Science.

[89] Kwun H.J., Guastafierro A., Shuda M., Meinke G., Bohm A., Moore P.S., Chang Y. (2009). The minimum replication origin of merkel cell polyomavirus has a unique large T-antigen loading architecture and requires small T-antigen expression for optimal replication. J. Virol..

[90] Houben R., Shuda M., Weinkam R., Schrama D., Feng H., Chang Y., Moore P.S., Becker J.C. (2010). Merkel cell polyomavirus-infected Merkel cell carcinoma cells require expression of viral T. antigens. J. Virol..

[91] Borchert S., Czech-Sioli M., Neumann F., Schmidt C., Wimmer P., Dobner T., Grundhoff A., Fischer N. (2014). High-affinity Rb binding, p53 inhibition, subcellular localization, and transformation by wild-type or tumor-derived shortened Merkel cell polyomavirus large T. antigens. J. Virol..

[92] Vassilev L.T. (2007). MDM2 inhibitors for cancer therapy. Trends Mol. Med..

[93] Kallen J., Goepfert A., Blechschmidt A., Izaac A., Geiser M., Tavares G., Ramage P., Furet P., Masuya K., Lisztwan J. (2009). Crystal Structures of Human MdmX (HdmX) in Complex with p53 Peptide Analogues Reveal Surprising Conformational Changes. J. Biol. Chem..

[94] Yee-Lin V., Pooi-Fong W., Soo-Beng A.K. (2018). Nutlin-3, A p53-Mdm2 Antagonist for Nasopharyngeal Carcinoma Treatment. Mini Rev. Med. Chem..

[95] Vassilev L.T., Vu B.T., Graves B., Carvajal D., Podlaski F., Filipovic Z., Kong N., Kammlott U., Lukacs C., Klein C. (2004). In vivo activation of the p53 pathway by small-molecule antagonists of MDM2. Science.

[96] Apontes P., Leontieva O.V., Demidenko Z.N., Li F., Blagosklonny M.V. (2011). Exploring long-term protection of normal human fibroblasts and epithelial cells from chemotherapy in cell culture. Oncotarget.

[97] Voon Y.L., Ahmad M., Wong P.F., Husaini R., Ng W.T.-W., Leong C.-O., Lane D.P., Khoo A.S.-B. (2015). Nutlin-3 sensitizes nasopharyngeal carcinoma cells to cisplatin-induced cytotoxicity. Oncol. Rep..

[98] Renouf B., Hollville E., Pujals A., Tetaud C., Garibal J., Wiels J. (2009). Activation of p53 by MDM2 antagonists has differential apoptotic effects on Epstein-Barr virus (EBV)-positive and EBV-negative Burkitt's lymphoma cells. Leukemia.

[99] Forte E., Luftig M.A. (2009). MDM2-dependent inhibition of p53 is required for Epstein-Barr virus B-cell growth transformation and infected-cell survival. J. Virol..

[100] Sarek G., Kurki S., Enback J., Iotzova G., Haas J., Laakkonen P., Laiho M., Ojala P.M. (2007). Reactivation of the p53 pathway as a treatment modality for KSHV-induced lymphomas. J. Clin. Investig..

[101] Sarek G., Ojala P.M. (2007). p53 reactivation kills KSHV lymphomas efficiently in vitro and in vivo: New hope for treating aggressive viral lymphomas. Cell Cycle.

[102] Petre C.E., Sin S.H., Dittmer D.P. (2007). Functional p53 signaling in Kaposi's sarcoma-associated herpesvirus lymphomas: Implications for therapy. J. Virol..

[103] Burgess A., Chia K.M., Haupt S., Thomas D., Haupt Y., Lim E. (2016). Clinical Overview of MDM2/X.-Targeted Therapies. Front Oncol..

[104] Carry J.C., Garcia-Echeverria C. (2013). Inhibitors of the p53/hdm2 protein-protein interaction-path to the clinic. Bioorg. Med. Chem. Lett..

[105] Lemos A., Leao M., Soares J., Palmeira A., Pinto M., Saraiva L., Sousa M.E. (2016). Medicinal Chemistry Strategies to Disrupt the p53-MDM2/MDMX Interaction. Med. Res. Rev..

[106] Bernal F., Wade M., Godes M., Davis T.N., Whitehead D.G., Kung A.L., Wahl G.M., Walensky L.D. (2010). A stapled p53 helix overcomes HDMX-mediated suppression of p53. Cancer Cell.

[107] Carvajal L.A., Neriah D.B., Senecal A., Benard L., Thiruthuvanathan V., Yatsenko T., Narayanagari S.-R., Wheat J.C., Todorova T.I., Mitchell K. (2018). Dual inhibition of MDMX and MDM2 as a therapeutic strategy in leukemia. Sci. Transl. Med..

[108] Ng S.Y., Yoshida N., Christie A.L., Ghandi M., Dharia N.V., Dempster J., Murakami M., Shigemori K., Morrow S.N., Scoyk A. (2018). Targetable vulnerabilities in T- and NK-cell lymphomas identified through preclinical models. Nat. Commun..

[109] Schiffman M., Doorbar J., Wentzensen N., De Sanjosé S., Fakhry C., Monk B.J., Stanley M.A., Franceschi S. (2016). Carcinogenic human papillomavirus infection. Nat. Rev. Dis. Primers.

[110] Vande Pol S.B., Klingelhutz A.J. (2013). Papillomavirus E6 oncoproteins. Virology.

[111] Zanier K., Stutz C., Kintscher S., Reinz E., Sehr P., Bulkescher J., Hoppe-Seyler K., Trave G., Hoppe-Seyler F. (2014). The E6AP binding pocket of the HPV16 E6 oncoprotein provides a docking site for a small inhibitory peptide unrelated to E6AP, indicating druggability of E6. PLoS ONE.

[112] Stutz C., Reinz E., Honegger A., Bulkescher J., Schweizer J., Zanier K., Travé G., Lohrey C., Hoppe-Seyler K., Hoppe-Seyler F. (2015). Intracellular Analysis of the Interaction between the Human Papillomavirus Type 16 E6 Oncoprotein and Inhibitory Peptides. PLoS ONE.

